# *Gss* deficiency causes age-related fertility impairment via ROS-triggered ferroptosis in the testes of mice

**DOI:** 10.1038/s41419-023-06359-x

**Published:** 2023-12-19

**Authors:** Haixia Zhu, Yin Cheng, Xianmei Wang, Xing Yang, Min Liu, Jun Liu, Shuqiao Liu, Hongxiang Wang, Aizhen Zhang, Runze Li, Chao Ye, Jian Zhang, Jiangang Gao, Xiaolong Fu, Bin Wu

**Affiliations:** 1 Department of Pharmacology, School of Pharmaceutical Sciences, Cheeloo College of Medicine, Shandong University, Jinan, 250100 China; 2 School of Life Science and Key Laboratory of the Ministry of Education for Experimental Teratology, Shandong University, Jinan, 250100 China; 3 Department of Reproductive Medicine, Central Hospital Affiliated to Shandong First Medical University, Jinan, 250013 China; 4 Medical Science and Technology Innovation Center, Shandong First Medical University, Jinan, 250117 China; 5 Shandong Aimeng Biological Technology Co., Ltd, Jinan, 250023 China; 6 Cheeloo College of Medicine, Shandong University, Jinan, 250012 China

**Keywords:** Developmental biology, Molecular biology, Urogenital reproductive disorders

## Abstract

Glutathione synthetase (GSS) catalyzes the final step in the synthesis of glutathione (GSH), a well-established antioxidant. Research on the specific roles of the *Gss* gene during spermatogenesis remains limited due to the intricate structure of testis. In this study, we identified pachytene spermatocytes as the primary site of GSS expression and generated a mouse model with postnatal deletion of *Gss* using Stra8-Cre (S8) to investigate the role of GSS in germ cells. The impact of *Gss* knockout on reducing male fertility is age-dependent and caused by ferroptosis in the testis. The 2-month-old S8/*Gss*^−/−^ male mice exhibited normal fertility, due to a compensatory increase in GPX4, which prevented the accumulation of ROS. With aging, there was a decline in GPX4 and an increase in ALOX15 levels observed in 8-month-old S8/*Gss*^−/−^ mice, resulting in the accumulation of ROS, lipid peroxidation, and ultimately testicular ferroptosis. We found that testicular ferroptosis did not affect spermatogonia, but caused meiosis disruption and acrosome heterotopia. Then the resulting aberrant sperm showed lower concentration and abnormal morphology, leading to reduced fertility. Furthermore, these injuries could be functionally rescued by inhibiting ferroptosis through intraperitoneal injection of GSH or Fer-1. In summary, *Gss* in germ cells play a crucial role in the resistance to oxidative stress injury in aged mice. Our findings deepen the understanding of ferroptosis during spermatogenesis and suggest that inhibiting ferroptosis may be a potential strategy for the treatment of male infertility.

## Facts and questions

- Conditional deletion of *Gss* decreases the fertility of 8-month-old male mice
- Disrupted meiosis and ectopic acrosome are observed in 8-month-old S8/*Gss*^−/−^ mice
- *Gss* knockout in germ cells causes testis ferroptosis in older mice
- Both GSH and Fer-1 improve the fertility of 8-month-old S8/*Gss*^−/−^ male mice

## Introduction

Male infertility is a multifactorial pathological condition that affects approximately 7% of the male population, and is primarily attributed to impaired spermatogenesis [1, 2]. Spermatogenesis begins with spermatogonia cells derived from spermatogonial stem cells, and progresses from the basal lamina towards the lumen of the seminiferous tubules [3]. Spermatogonia differentiate into spermatocytes, and then spermatocytes meiosis into round spermatids. There are unique processes occur during meiosis, including programmed double-stranded DNA breaks, meiotic recombination, and any of these mechanisms function improperly, it can result in meiosis arrest. The process of meiotic division produces haploid spermatids, which are initially round and become elongated via acrosome formation, nuclear chromatin condensation, mitochondrial rearrangement, flagellum biogenesis and the removal of unnecessary cytoplasm [4]. Spermatogenesis is influenced by various factors or harmful substances and in which multiple signaling pathways are involved [5–7]. Age is one of the relevant factors, it was showed by the alters of spermatogenic dynamics and the decreases of sperm quality in aging men [8, 9]. The fecundity reduction with aging is referred as age-related fertility decline, and accumulation of oxidative damage and DNA fragmentation in cells and tissues plays a prominent role in this process [8, 10, 11]. It has been reported that advancing age increases sperm chromatin damage and decreases fertility in peroxiredoxin 6 knockout mice [12]. Various cell death processes are also involved in spermatogenesis, such as apoptosis and autophagy, and recently, a new form of regulated cell death, ferroptosis, has been reported to be involved in spermatogenesis [13–15].

Ferroptosis is an iron-dependent cell death mainly characterized by intracellular iron accumulation, lipid peroxidation, and mitochondrial damage [16]. Numerous organ damage and degenerative diseases are driven by iron death, including cancer, ischemic organ damage, neurodegeneration, hepatopulmonary fibrosis, and autoimmune diseases [17]. Excessive oxidation of Fe^2+^ with peroxide groups and harmful products from the oxidation reaction can damage the integrity and stability of cell membranes [18]. Since sperm cell membranes are rich in polyunsaturated fatty acids (PUFAs), they are highly susceptible to ROS attack and lipid peroxidation [19]. However, the impact of ferroptosis on spermatogenesis and male fertility remains poorly understood.

Glutathione (GSH) is a potent antioxidant, and is oxidized to GSSG in the presence of glutathione peroxidase 4 (GPX4), thereby reduces peroxides to the corresponding alcohols to reduce the generation of toxic free radicals and inhibit lipid peroxidation [20]. GSH exerts beneficial effects on sperm and plays a crucial role in the process of ferroptosis. GSH synthetase (GSS) is an enzyme that catalyzes the second step in synthesis of GSH. Although overexpression of *Gss* failed to increase GSH level, *Gss* is important for determining the overall GSH synthesis capacity in certain tissues or under stress conditions [21–23]. *GSS*-deficient patients exhibit significant metabolic consequences since the conversion of accumulated γ-glutamylcysteine to 5-oxo-proline can cause severe metabolic acidosis, hemolytic anemia, and central nervous system damage [24, 25]. Approximately 25% of patients with *GSS* deficiency die from infections and electrolyte imbalances in the neonatal period [26]. The role and mechanism of GSS in spermatogenesis remain unclear.

In the study, we demonstrated the role of GSS/GSH in male germ cells though conditional deletion of *Gss* mice via Stra8-Cre. Additionally, we supplemented the mice with GSH and ferrostatin-1 (Fer-1) to alleviate the testicular injury caused by *Gss* deficiency and improve their fertility.

## Results

### ROS level was increased in testis and sperms of aged mice

We firstly analyzed the oxidative stress level and sperm quality in mouse testis as a function of age. The levels of GSH and ROS in testes of WT mice at different ages were examined to test the oxidative stress level. We discovered that the GSH levels were decreased in the testis of 16-month-old mice (Fig. S1A). Consistent with this, the mRNA and protein levels of *Gss* have no difference between 2 months and 8 months age, and decreased significantly in the testis of 16-month-old mice (Fig. S1B, C). Additionally, compared with those in 2-month-old mice, the ROS levels in testis and sperms at 16-month-old mice were increased (Fig. S1D, E). H&E staining of testicular sections demonstrated that the testicular structure of 16-month-old mice was disorganized (presented in the red dashed box) and vacuolation appeared (presented in the black dashed box) (Fig. S1F). The sperm concentration in cauda epididymis was decreased in 16-month-old mice (Fig. S1F, H). The percentage of abnormal sperms was also increased, including sperms with abnormal heads, coiled tails, and decapitated sperms (Fig. S1G, I, J). The CASA analysis demonstrated that sperm motility in 16-month-old mice also decreased (Fig. S1K). Compared with those in 2-month-old mice, the ROS level was not increased and the testis and sperm quality did not change in 8-month-old WT mice (Fig. S1D, K). Decreased sperm quality accompanied by an imbalance of GSH and ROS levels in 16-month-old mice suggested that sperm quality in aged mice may be closely related to oxidative stress levels.

### Knockout of *Gss* in germ cells reduced male fertility in 8-month-old mice

**Fig. 1:**
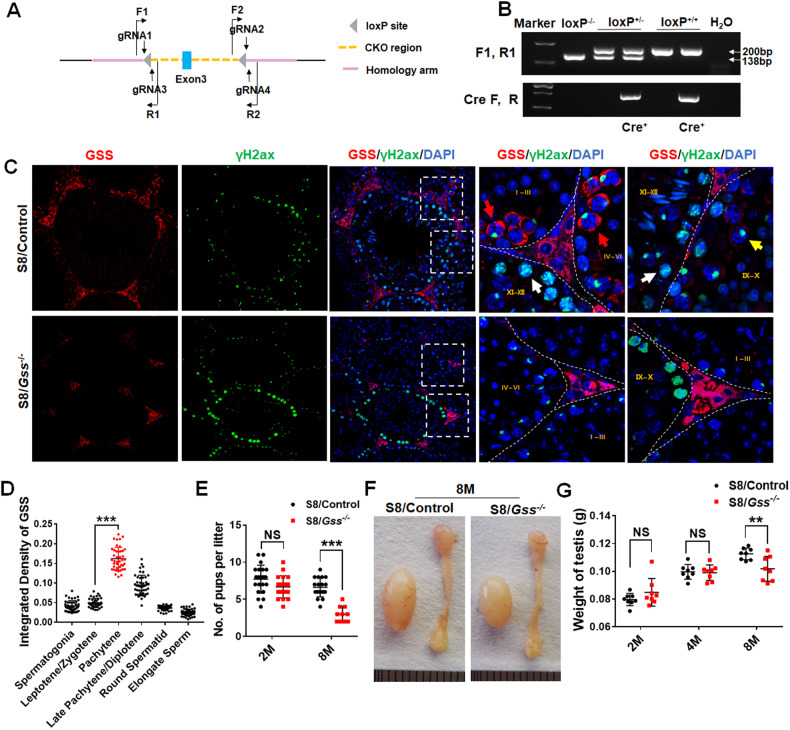
Impact of *Gss* deficiency in germ cells on fertility. **A** Schematic diagram of generating a *Gss*-Flox mouse model by CRISPR/Cas9 system. **B** Agarose gel analysis of PCR products amplified used primer F1, R1, and Cre F, R. Wild-type allele: one band with 138 bp; Heterozygotes: two bands with 200 bp and 138 bp; Homozygotes: one band with 200 bp. **C** Testicular sections of S8/Control and S8/*Gss*^*−/−*^ mice for *Gss* (red), γH2ax (green), and DAPI (blue) staining. The two columns on the right are enlarged images in a white dotted box. Scar bar = 50 μm. **D** The statistic for fluorescence intensity of GSS in various germ cells. ***P < *0.001*; more than 40 cells were counted from 4 biologically independent animals for every group. **E** Litter size of 2- and 8-month-old S8/Control and S8/*Gss*^−/−^ male mice that were caged with WT females for 3 months. ***P < *0.001*; NS indicates non-significant, *n* = 4 (biologically independent animals). **F** The testes of S8/*Gss*^*−/−*^ mice were smaller than those of S8/Control mice at 8 months of age. **G** The testicular weight of the S8/Control and S8/*Gss*^*−/−*^ mice, at 2, 4, and 8 months. **P < *0.01*; NS indicates non-significant; *n* = 8 (testes from eight different animals).

GSH, as a scavenger of ROS, is synthesized through a two-step process, with the final step catalyzed by GSS. To investigate the role of GSS in male reproduction, we constructed *Gss* ^*Flox/Flox*^ mice by inserting two LoxP site to the left and right of exon 3 (Fig. 1A). Sequencing results of homozygous mice confirmed the successful insertion of both LoxP sites (Fig. S2A, B). Stra8-Cre expression begins at 3 days postnatally and initiates meiosis via type A1 spermatogonia [27, 28]. The germ cell-specific *Gss* knockout mouse was generated by crossing the *Gss* ^*Flox/Flox*^ and Stra8*-*Cre. Agarose gel analysis of PCR products was used to identify genotype. The heterozygous genotype exhibited two bands, whereas the homozygous mutation indicated only one 200-bp band (Fig. 1B). We referred to the homozygous mutants with Stra8-Cre as S8/*Gss*^−/−^ and their littermate controls as S8/Control. In the testis, GSS is abundantly expressed in Leydig cells (Fig. 1C). According to the expression pattern of γH2AX in the testis [29, 30], the fluorescence intensity of GSS expression in various germ cells was counted using ImageJ software. In S8/Control male mice, GSS was at a lower level in spermatogonia, leptotene, and zygotene spermatocytes (Fig. 1C,D). It was increased significantly in early/mid pachytene spermatocytes in seminiferous tubules before stage VII–VIII (red arrows) and reduced in late pachytene and diplotene spermatocytes in the seminiferous tubules after stage IX–X (yellow arrows), finally returned to a low level at diplotene spermatocytes, round spermatids, and elongated sperms (white arrows) (Fig. 1C, D). GSS was not detected in germ cells in S8/*Gss*^−/−^ mice, indicating that the specific knockout model of *Gss* was successfully constructed (Fig. 1C). The fertility of male mice was tested by mating two 2-month WT female mice over 3 months. There was no difference in litter size between 2-month-old S8/Control and S8/*Gss*^−/−^ male mice; however, in the 8-month-old group, the litter size of S8/*Gss*^−/−^ males was significantly smaller than in the S8/Control males (Fig. 1E). The testicular volume and weight of 8-month S8/*Gss*^−/−^ males were smaller than those of S8/Control mice, whereas those in 2- and 4-month-old mice presented no difference (Fig. 1F, G).

### The germ cells were decreased in the testes of 8-month-old S8/*Gss*^−/−^ mice

**Fig. 2:**
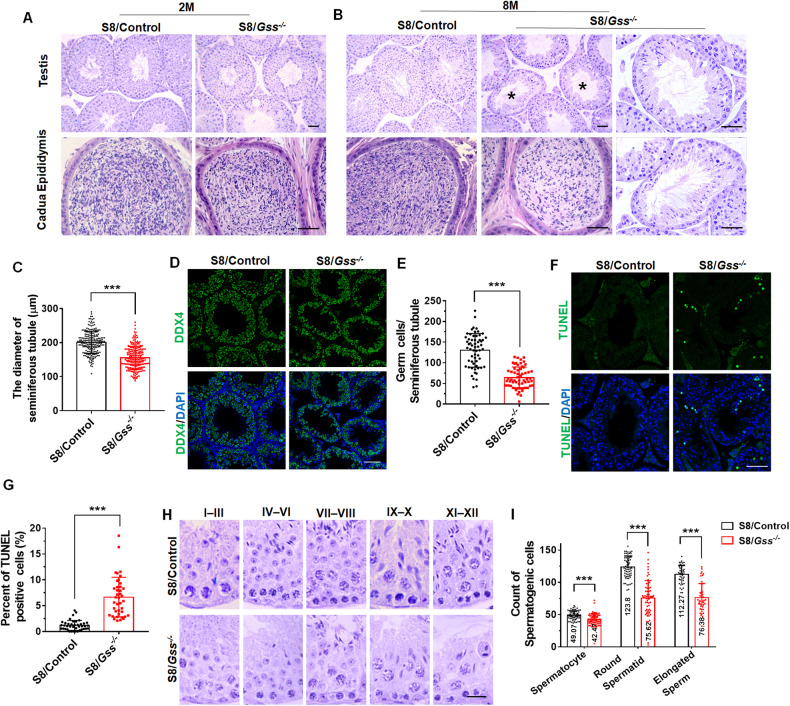
The deletion of *Gss* in germ cells caused abnormal spermatogenesis. **A** The H&E staining of testis and caudal epididymis from 2-month-old S8/Control and S8/*Gss*^−/−^ mice. Scar bar = 50 μm. **B** Histological structures of testis and caudal epididymis in 8-month-old S8/Control and S8/*Gss*^−/−^ mice. The images in the far-right column are the magnified view of the seminiferous tubules labeled with an asterisk. Scar bars = 50 μm. **C** The diameters of the seminiferous tubules were measured in 8-month S8/Control and S8/*Gss*^−/−^ mice. ***P < *0.001*, 240 seminiferous tubules were counted for each group. **D** The immunofluorescence staining of DDX4 in testes from 8-month-old S8/Control and S8/*Gss*^−/−^ mice. Scar bar = 50 μm. **E** Germ cell quantification in the single seminiferous tubule of 8-month-old mice. ***P < *0.001*; *n* = 6 (biologically independent animals); 10 tubules were tested for each mouse. **F, G** TUNEL staining and the percentage of positive cells in the testicular sections from 8-month-old S8/Control and S8/*Gss*^−/−^ mice. Scar bar = 50 μm. ***P < *0.001*; *n* = 4 (biologically independent animals); more than 10 apoptotic tubules were counted for each mouse. **H** The H&E staining of the seminiferous tubules at different stages. Scar bar = 20 μm. **I** The count of spermatogenic cells in 8-month-old S8/Control and S8/*Gss*^−/−^ mice. ***P < *0.001*; *n* = 6 (biologically independent testes from six different animals); more than 65 tubules were counted for spermatocytes and round spermatids, and 45 tubules at stages IX–X were used to analyze elongated sperms.

We further examined the tissue structure of mouse testis. In the testes of 2-month-old S8/*Gss*^−/−^ mice, the structure of seminiferous tubules was arranged with occasional vacuoles (Fig. 2A). Its cauda epididymis was not different from that of S8/Control mice (Fig. 2A). When compared with that in S8/Control mice, the arrangement of germ cells was sparse and disordered and a large number of germ cells were lost in the seminiferous tubules of 8-month S8/*Gss*^−/−^ mice (Fig. 2B). Next, we observed the testes of 8-month-old mice. We counted 240 seminiferous tubules and measured their diameter using a microscope; the diameter of seminiferous tubules in 8-month S8/*Gss*^−/−^ mice was lower than that in 8-month S8/Control mice (Fig. 2C). Staining results of DDX4 (germ cell-specific marker) demonstrated a decrease in the number of germ cells in 8-month S8/*Gss*^−/−^ mice (Fig. 2D,E). TUNEL-positive signals that mark DNA fragmentation are often detected in testes with the loss of germ cells [31, 32]. TUNEL results presented that DNA damage was increased in 8-month-old S8/*Gss*^−/−^ mice (Fig. 2F,G). We distinguished spermatogenic stages by H&E staining. It was observed that germ cells were sparsely arranged in the testes of S8/*Gss*^−/−^ mice compared with those in the testes of S8/Control mice (Fig. 2H). The apparent decline in the number of spermatocytes, round spermatids, and elongated sperm suggested that spermiogenesis was disrupted in the testis of 8-month S8/*Gss*^−/−^ mice (Fig. 2I).

### Meiosis was disrupted in 8-month-old S8/*Gss*^−/−^ mice

**Fig. 3:**
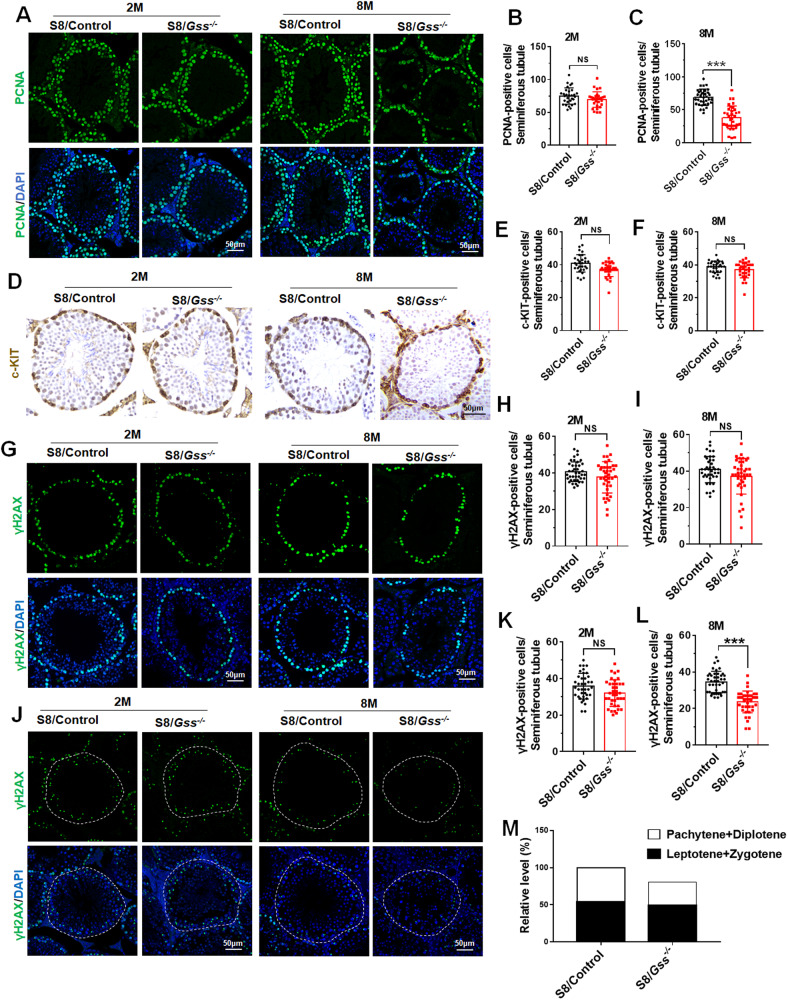
*Gss* deficiency affects meiosis during spermatogenesis. **A**–**C** Immunofluorescence staining of PCNA in testicular sections from 2- and 8-month-old mice. Scar bar = 50 μm. ***P < *0.001*; NS indicates non-significant, *n* = 3 (biologically independent animals); more than ten tubules were counted for every mouse. **D**–**F** The immunohistochemical assay for c-KIT was used to examine spermatogonia differentiation. Scar bar = 50 μm. NS indicates non-significant, *n* = 3 (biologically independent animals); more than ten tubules were counted for every mouse. **G**–**I** Immunofluorescence detection of γH2AX and the statistical results in testis from S8/Control and S8/*Gss*^−/−^ mice at 2 or 8 months. **J**–**L** The test and count results of spermatocytes with punctate γH2AX expression in the testes of 2- and 8-month-old mice. **G**–**L** Scar bar = 50 μm. ***P < *0.001*; NS indicates non-significant, *n* = 4 (biologically independent animals); more than ten tubules were counted for every mouse. **M** Diagram of the relative number of germ cells in the testis of 8-month-old S8/Control and S8/*Gss*^−/−^ mice.

To detect which spermatogenic stage was blocked, we tested the proliferation of spermatogonia and spermatocyte by PCNA (a marker for cell proliferation) staining. No change was observed in the testes of 2-month-old S8/*Gss*^−/−^ mice (Fig. 3A, B). In the 8-month-old S8/*Gss*^−/−^ mice, the number of PCNA-positive germ cells in individual seminiferous tubules was significantly decreased (Fig. 3A, C). The c-KIT was used to examine spermatogonia differentiation, and we counted the number of c-KIT-positive cells in the seminiferous tubules in which spermatogonia differentiation was active. Compared with that in S8/Control mice, there was no difference in differentiated spermatogonia in both 2- and 8-month-old S8/*Gss*^−/−^ mice (Fig. 3D–F). This indicated that the number of spermatogonia was not affected by *Gss* knockout. γH2AX is a sensitive molecular marker of DNA damage and repair and was used to test spermatocytes at different stages during spermatogenesis [33, 34]. At the initiation of meiosis, γH2AX was abundantly expressed in spermatocytes at the periphery of seminiferous tubules, as indicated in S8/Control mice (Fig. 3G). The number of spermatocytes at this stage did not change in both 2- and 8-month-old S8/*Gss*^−/−^ mice (Fig. 3H, I). During the pachytene stage, γH2AX was resolved by homologous recombination and remained on XY bodies in a punctate manner. The number of spermatocytes expressing punctate γH2AX was decreased in the testes of 8-month-old S8/*Gss*^−/−^ mice (Fig. 3J, L), and this was not observed in 2-month-old mice (Fig. 3J, K). Figure 3M summarizes the effect of *Gss* deficiency on meiosis in 8-month-old mice; the decrease in spermatocyte number was mainly caused by the decrease of spermatocytes in the pachytene and diplotene stages. These results suggested that *Gss* knockout in germ cells impaired spermatocyte meiosis progression.

**Fig. 4:**
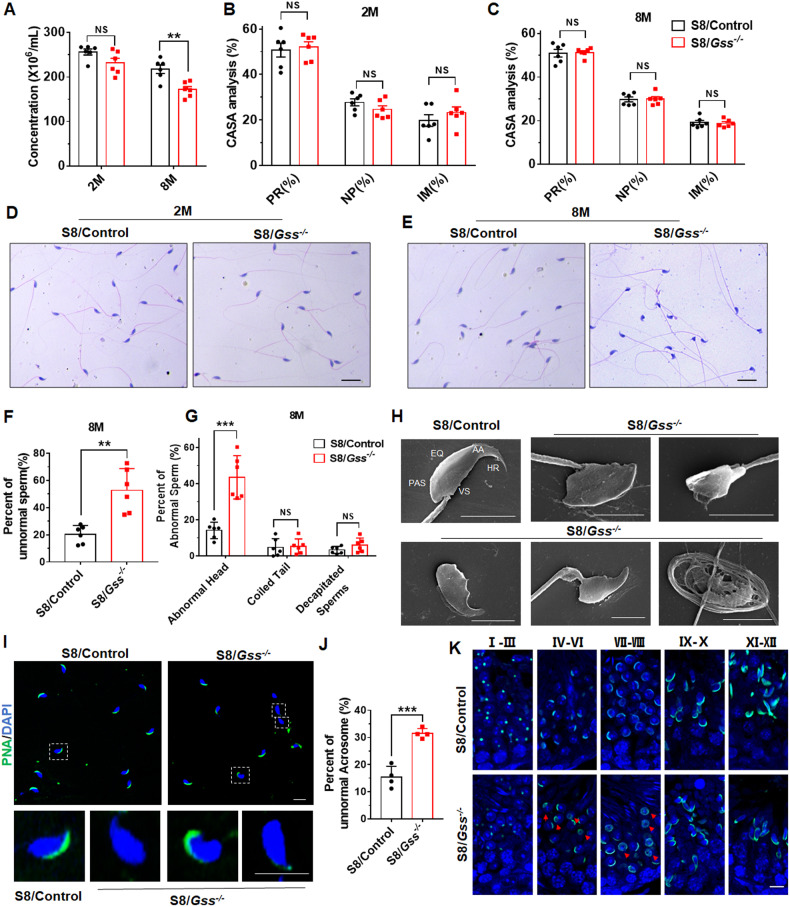
Analysis of sperms from 2- and 8-month-old mice. **A** Sperm concentration in cauda epididymis of S8/Control and S8/*Gss*^−/−^ mice at 2 and 8 months of age under the same treatment condition. NS indicates non-significant, **P < *0.01*; *n* = 6 (biologically independent animals). **B**, **C** CASA analysis of sperms from cauda epididymis of 2- and 8-month-old mice. NS indicates non-significant, *n* = 6 (biologically independent animals). **D**, **E** The H&E staining of sperms from cauda epididymis of S8/Control and S8/*Gss*^−/−^ mice at 2 or 8 months. Scar bar = 20 μm. **F** The proportion of malformed sperms in 8-month-old mice, and **G** the proportion of different types of malformed sperms. **P < *0.01*, ***P < *0.001*; NS indicates non-significant, *n* = 6 (biologically independent animals). **H** SEM images of sperms from 8-month-old S8/Control and S8/*Gss*^−/−^ mice. Scar bar = 5 μm. PAS post acrosomal segment, EQ equatorial segment, AA anterior acrosome, HR hook rim, VS ventral spur. **I** The PNA staining of sperms of 8-month-old S8/Control and S8/*Gss*^−/−^ mice, and **J** the proportion of abnormal morphological acrosomes. The images on the bottom are the enlarged view in the white dotted boxes. Scar bar = 10 μm. ***P < *0.001*; *n* = 4 (biologically independent animals); more than 200 sperms were counted for each mouse. **K** The PNA staining of testicular slices was used to examine acrosome development in mice. The red arrow points to the abnormally located acrosome. Scar bar = 10 μm.

### Acrosome abnormality were observed in 8-month-old S8/*Gss*^−/−^ mice

We examined the sperm quality from cauda epididymis of mice aged 2 and 8 months. Under the same condition, there was no difference in sperm concentration between S8/Control and S8/*Gss*^−/−^ mice at 2 months of age, albeit the sperm concentration of S8/*Gss*^−/−^ mice was significantly decreased at 8 months of age (Fig. 4A). The CASA analysis demonstrated that sperm motility did not change in cauda epididymis of 2- or 8-month-old S8/Control and S8/*Gss*^−/−^ mice (Fig. 4B, C). This suggested that *Gss* knockout in germ cells did not affect sperm motility. Next, H&E staining was performed to sperm smears. The rate of deformed sperms from S8/*Gss*^−/−^ mice was similar to that from S8/Control mice at 2 months of age (Fig. 4D and Fig. S3A), and the proportion of various types of malformations was also similar (Fig. S3B). However, the proportion of sperms with abnormal morphology was increased in S8/*Gss*^−/−^ mice after birth for 8 months (Fig. 4E,F), and sperm head deformities were particularly severe (Fig. 4G). SEM was used to intuitively observe the morphology of the sperm head. The typical structures of sperm heads observed in S8/Control mice were absent in 8-month-old S8/*Gss*^−/−^ mice (Fig. 4H). Acrosomes play a crucial role in successful fertilization, and FITC-labeled PNA was used to examine sperm acrosomes. The results demonstrated a significant increase in the percentage of sperms with abnormal acrosomes in 8-month-old S8/*Gss*^−/−^ mice, including no acrosome, deformed acrosome, and acrosomal diffusion (Fig. 4I,J). PNA staining for the testis revealed that normal acrosome development undergoes typical point, cap-shaped, and crescent-shaped and further stretching deformation. In 8-month-old S8/*Gss*^−/−^ mice, ectopic expression of the acrosome occurs at the cap-shaped acrosome and crescent-shaped acrosome stages (Fig. 4K). These results suggested that *Gss* deficiency impaired the development of acrosome in 8-month-old mice.

### *Gss* knockout increased autophagy in mouse testis

**Fig. 5:**
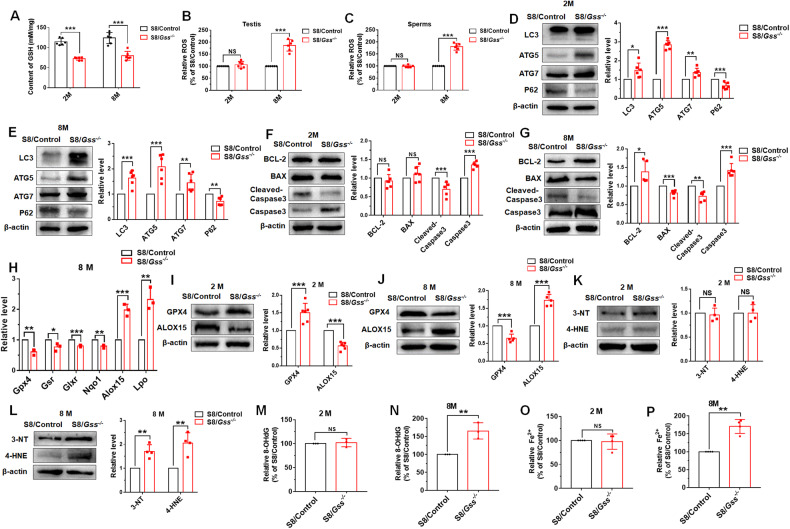
Ferroptosis were observed in the testes of 8-month-old S8/*Gss*^−/−^ mice. **A** The content of GSH in testis from S8/Control and S8/*Gss*^−/−^ mice at 2 or 8 months. **B**, **C** The relative levels of ROS in testis and sperms from 2- or 8-month-old S8/Control and S8/*Gss*^−/−^ mice. **A**–**C** ***P < *0.001*; NS indicates non-significant, *n* = 6 (biologically independent animals). **D**, **E** The expression levels of autophagy-related proteins, including LC3, ATG5, ATG7, and P62, in the testis of 2- or 8-month-old mice. *P < *0.05*, **P < *0.01*, ***P < *0.001*; *n* = 6 (biologically independent animals). **F**, **G** Western blotting to determine the apoptosis-related proteins levels, including BCL-2, BAX, cleaved-Caspase 3, and Caspase 3. *P < *0.05*, **P < *0.01*, ***P < *0.001*; NS indicates non-significant, *n* ≥ 5 (biologically independent animals). **H** The mRNA levels of anti-oxidant genes (*Gpx4, Gsr*, *Glrx*, *Nqo1*) and pro-oxidation genes (*Alox15, Lpo*) were examined in the testes of 8-month-old S8/Control and S8/*Gss*^−/−^ mice. *P < *0.05*, **P < *0.01*, ***P < *0.001*; *n* = 3 (biologically independent animals). **I** The protein levels of GPX4 and ALOX15 in the testis were examined in 2-month-old mice. ***P < *0.001*; *n* = 6 (biologically independent animals). **J** The GPX4 and ALOX15 expressions were determined in testis from 8-month-old S8/Control and S8/*Gss*^−/−^ mice by western blot. ***P < *0.001*; *n* = 6 (biologically independent animals). **K** Western blot was used to determine the levels of 3-NT and 4-HNE in the testes of 2-month-old mice. NS indicates non-significant; *n* = 4 (biologically independent animals). **L** The levels of 3-NT and 4-HNE in the testes were increased in 8-month-old S8/*Gss*^−/−^ mice compared to those in S8/Control mice. **P < *0.01,* *n* = 4 (biologically independent animals). **M**, **O** The relative levels of 8-OHdG and Fe^2+^ exhibited no differences between 2-month-old S8/Control and S8/*Gss*^−/−^ mice. NS means non-significant, n = 3 (**J**) and 4 (**L**) biologically independent animals. **N**, **P** The relative levels of 8-OHdG and Fe^2+^ of the testes of 8-month-old mice were determined by the corresponding assay kit. **P < *0.01*; *n* = 3 (**N**) and 4 (**P**) (biologically independent animals).

We further explored the molecular mechanisms which *Gss* affects fertility in male mice. The levels of GSH were significantly decreased in the testes of both 2- and 8-month-old S8/*Gss*^−/−^ mice (Fig. 5A). GSH is considered the scavenger of ROS [35], and thus, the ROS levels in testis and sperms were tested. Compared with that in S8/Control mice, there were no changes in ROS level in 2-month S8/*Gss*^−/−^ mice. However, the ROS levels of both testis and sperms were increased in 8-month S8/*Gss*^−/−^ mice (Fig. 5B,C). ROS and autophagy are closely associated in two ways—induction of autophagy by oxidative stress and reduction of ROS by autophagy [36]. In the testes of 2-month-old S8/*Gss*^−/−^ mice, the protein expression levels of autophagy-related proteins LC3, ATG5, and ATG7 were significantly increased and P62 expression was decreased compared to those in S8/Control mice (Fig. 5D). It was identical to the expression of autophagy-related proteins in the testes of 8-month-old mice (Fig. 5E). This suggested that *Gss* knockout activated autophagy, while autophagy activation is not the cause of the difference in male fertility between 2- and 8-month-old S8/*Gss*^−/−^ mice. Additionally, there was no significant change in the anti-apoptotic protein Bcl-2 and pro-apoptotic protein Bax; however, Caspase 3 expression was increased and cleaved-Caspase 3 level was decreased in the testes of 2-month S8/*Gss*^−/−^ mice (Fig. 5F). In the testes of 8-month S8/*Gss*^−/−^ mice, the apoptosis-related signaling pathway was inhibited, as evidenced by elevated Bcl-2 expression, decreased Bax level, and reduced Caspase 3 activity (Fig. 5G). Expression of Caspase 3 was elevated in both 2- and 8-month-old S8/*Gss*^−/−^ mice, albeit active cleaved-Caspase 3 levels were inhibited since cleavage of Caspase 3 requires the involvement of GSH [37].

### *Gss* deficiency caused age-related ferroptosis in the testis of mice

These results suggested that the focus should be on the ROS level, as the balance of anti-oxidant and pro-oxidant levels is closely related to ROS. The mRNA levels of the anti-oxidant genes (*Gpx4*, *Gsr*, *Glrx*, and *Nqo1*) decreased and those of the pro-oxidant genes (*Alox15* and *Lpo*) increased in the testes of S8/*Gss*^−/−^ mice at 8 months of age (Fig. 5H). Two of these proteins, GPX4 and ALOX15, which are associated with ferroptosis, were highlighted. At 2 months of age, compared with S8/Control mice, the protein expression of GPX4 was elevated and the level of ALOX15 was decreased in the testes of S8/*Gss*^−/−^ mice (Fig. 5I). GPX4 protein expression was significantly decreased whereas ALOX15 expression was significantly increased in the testes of 8-month-old S8/*Gss*^−/−^ mice (Fig. 5J). Ferroptosis is initiated by the inactivation of the GPX4 and exacerbated by the activity of ALOX15, accompanied by lipid peroxidation [38]. The versatile oxidative stress biomarker 3-nitrotyrosine (3-NT) and lipid peroxidation product 4-hydroxy-2-nonenal (4-HNE) revealed no differences between the testes of S8/Control and S8/*Gss*^−/−^ mice at 2 months of age (Fig. 5K). In the testes of 8-month-old S8/*Gss*^−/−^ mice, the levels of 3-NT and 4-HNE were significantly increased compared to those in the testes of S8/Control mice (Fig. 5L). Oxidative stress could cause the oxidative injury of DNA, and the oxidized DNA base compound 8-hydroxy-2′-deoxyguanosine (8‐OHdG) has extensively been used to reflect the degree of oxidative damage to DNA [39, 40]. Compared with that in S8/Control mice, the level of 8-OHdG did not change in the testes of 2-month-old S8/*Gss*^−/−^ mice (Fig. 5M) but elevated in the testes of 8-month-old S8/*Gss*^−/−^ mice (Fig. 5N). The level of Fe^2+^ in the testes of 2-month-old S8/*Gss*^−/−^ mice was similar to that of S8/Control mice (Fig. 5O) but elevated in the testes of 8-month-old S8/*Gss*^−/−^ mice (Fig. 5P). These results suggested that knockout of *Gss* in germ cells causes elevated levels of oxidative stress and lipid peroxidation in the testes of 8-month-old S8/*Gss*^−/−^ mice, thus, leading to testis ferroptosis.

### GSH and Fer-1 rescued testis injury and elevated sperm quality of S8/*Gss*^−/−^ mice

**Fig. 6:**
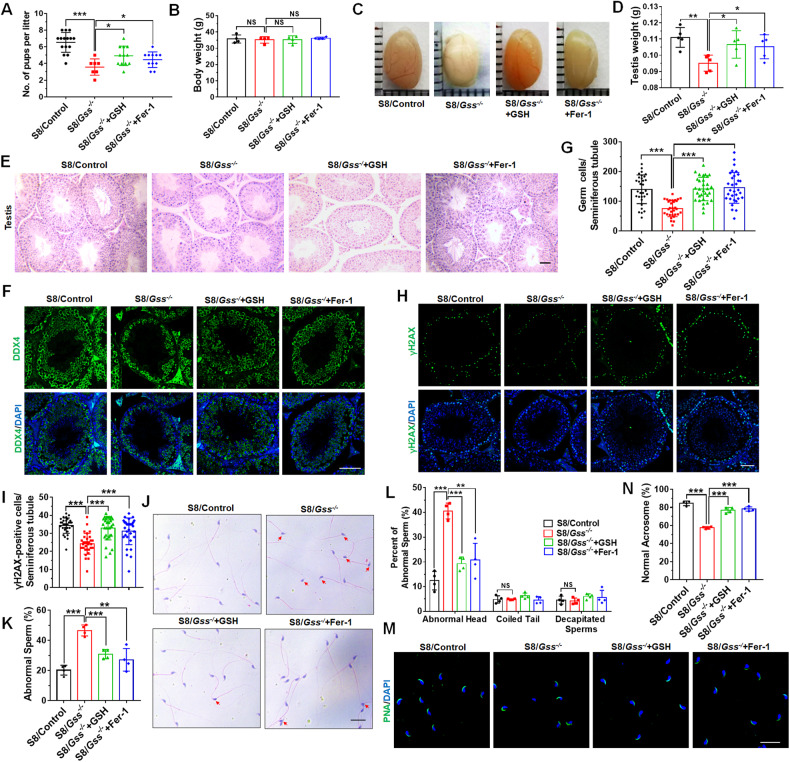
Effects of GSH and Fer-1 on 8-month-old S8/*Gss*^−/−^ mice. **A** Statistical results of litter size of mice after treatment with the GSH and Fer-1. *P < *0.05*, ***P < *0.001*, *n* = 3 (biologically independent male mice). **B** The body weight was recorded for mice in different groups. NS indicates non-significant. **C**, **D** The image and weight of the testes from S8/Control, S8/*Gss*^−/−^ mice, S8/*Gss*^−/−^ mice + GSH, and S8/*Gss*^−/−^ mice + Fer-1 mice. *P < *0.05*, **P < *0.01*; *n* = 5 (biologically independent animals). **E** The H&E staining of the testes of mice in different groups. Scar bar = 50 μm. **F** Immunofluorescence staining of DDX4 for testis. Scar bar = 50 μm. **G** The number of cells positive for DDX4 per seminiferous tubule. **H**, **I** Immunofluorescence staining of γH2AX and the statistical results of positive cells in tubules. **G**, **I** ***P < *0.001*, n = 3 (biologically independent animals); more than ten tubules were examined for each mouse. **J**, **K** The H&E staining image of sperms from cauda epididymis and the percentage of malformed sperms per the H&E staining results. Scar bar = 20 μm. **P < *0.01*, ***P < *0.001*; *n* = 4 (biologically independent animals). **L** Different types of abnormal sperms were counted, including abnormal heads, coiled tails, and decapitated sperms. **P < *0.01*, ***P < *0.001*; NS indicates non-significant, *n* = 4 (biologically independent animals). **M**, **N** The PNA was used to stain the acrosome of sperms for determining the percentage of sperms with normal acrosome. Scar bar = 20 μm. ***P < *0.001*, *n* = 4 (biologically independent animals); more than 200 sperms were counted for each mouse.

We explored whether 8-month-old S8/*Gss*^−/−^ mice can recover from testis injury; one way was to replenish GSH intraperitoneally, and another way was to inhibit ferroptosis in testes by injecting Fer-1 (ferroptosis inhibitor) intraperitoneally. After treating 7-month-old S8/*Gss*^−/−^ mice with GSH or Fer-1 for 4 weeks, WT females were caged with them. Treatment was continued for 8 weeks during caging, and litter size was recorded. The results demonstrated that both GSH and Fer-1 improved the fertility of S8/*Gss*^−/−^ mice (Fig. 6A). Following GSH or Fer-1 treatment, S8/*Gss*^−/−^ mice exhibited no changes in body weight (Fig. 6B) but had an increased testis volume and weight compared to S8/*Gss*^−/−^ mice without drugs (Fig. 6C,D). The H&E staining of testicular sections indicated an improvement in testicular tissue structure following GSH or Fer-1 treatment (Fig. 6E). Immunofluorescence staining and number counts of DDX4 revealed a significant increase in the number of germ cells in groups that received drug treatment (Fig. 6F, G). Both GSH and Fer-1 attenuated the meiotic arrest caused by the knockout of *Gss* in germ cells (Fig. 6H, I). Further, the number of sperms with abnormal morphology in the cauda epididymis was also reduced by GSH or Fer-1 (Fig. 6J, K), and the proportion of sperms with head malformation was significantly reduced in the S8/*Gss*^−/−^ mice after dosing (Fig. 6L). The proportion of sperms with normal morphology acrosome was also increased in the S8/*Gss*^−/−^ mice following treatment with GSH or Fer-1 (Fig. 6M, N). These results suggested that either GSH or Fer-1 can alleviate testis injury and improve sperm quality in older S8/*Gss*^−/−^ mice.

In addition, due to autophagy was activated in testes of 2- and 8-month-old S8/*Gss*^−/−^ mice, Bafilomycin A1 (an inhibitor of autophagy) was used to treat 7-month-old S8/*Gss*^−/−^ mice for 4 weeks. The results showed that the phenotypes of testis in S8/*Gss*^−/−^ mice were not alleviated (Fig. S4), including the weight and morphology of testis (Fig. S4A, B), the number of germ cells (Fig. S4C, D), and the percent of abnormal sperms (Fig. S4E–G).

### GSH and Fer-1 inhibited testis ferroptosis of older S8/*Gss*^−/−^ mice

**Fig. 7:**
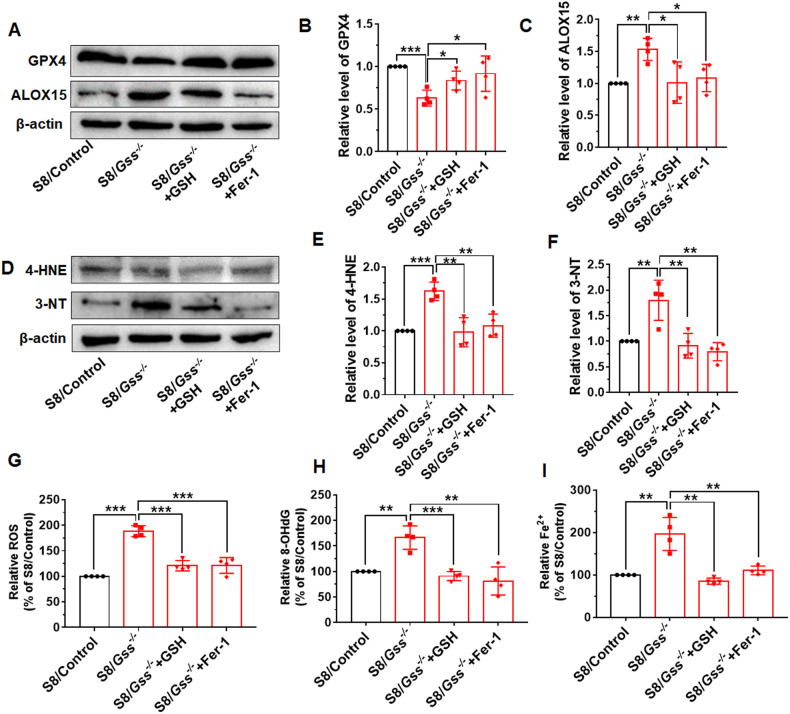
The ferroptosis levels were examined following GSH and Fer-1 treatment. **A**–**C** Western blot was used to detect the protein levels of GPX4 and ALOX15. *P < *0.05*, **P < *0.01*, ***P < *0.001*; *n* = 4 (biologically independent animals). **D**–**F** The lipid peroxidation markers 3-NT and 4-HNE in mice testis were determined by western blotting. **P < *0.01*, ***P < *0.001*, *n* = 4 (biologically independent animals). **G** The relative levels of ROS in testis of S8/Control, S8/*Gss*^−/−^ mice, S8/*Gss*^−/−^ mice + GSH, and S8/*Gss*^−/−^ mice + Fer-1 mice. **H** The contents of 8-OHdG of testis were examined by assay kit and normalized by S8/Control group. **I** The levels of Fe^2+^ were examined in the testis of mice treated with GSH and Fer-1. **G**–**I** *P < *0.05*, **P < *0.01*, ***P < *0.001*; *n* = 4 (biologically independent animals).

We further examined the effects of GSH and Fer-1 on testis ferroptosis. Western blot results revealed that compared with those in untreated S8/*Gss*^−/−^ mice, the protein expression of GPX4 was elevated and the protein level of ALOX15 was decreased in GSH and Fer-1-treated S8/*Gss*^−/−^ mice (Fig. 7A–C). The lipid peroxidation was reduced in the GSH or Fer-1-treated groups, indicated by the decreased levels of 4-HNE and 3-NT in the testes (Fig. 7D–F). The GSH and Fer-1 reduced the ROS level in the testes of S8/*Gss*^−/−^ mice (Fig. 7G), and compared with that in the S8/*Gss*^−/−^ mice, the relative level of 8-OHdG was also significantly decreased following drug administration (Fig. 7H). GSH and Fer-1 attenuated the oxidative stress and lipid peroxidation in the testes of S8/*Gss*^−/−^ mice. Moreover, the elevation of Fe^2+^ caused by deficiency of *Gss* in germ cells was also suppressed by both GSH and Fer-1 (Fig. 7I). These results indicated that GSH supplementation and Fer-1 play a protective role in the testis of older S8/*Gss*^−/−^ mice by inhibiting ferroptosis.

## Discussion

Ferroptosis is a recently discovered form of iron-dependent regulatory cell death, involving various diseases and playing an important role in the reproductive system and spermatogenesis [41]. GSH, as a potent anti-oxidant, can influence the ferroptosis process and participate in spermatogenesis [42, 43]. In this study, to investigate the function of *Gss*/GSH in germ cells, *Gss*-specific knockout mice were constructed using Stra8-cre mice. We discovered that 8-month-old S8/*Gss*^−/−^ male mice exhibited significantly reduced fertility. We analyzed the mechanism by which *Gss* deficiency reduced fertility in older male mice and identified molecular targets that may be used to attenuate testis injury.

The balance of antioxidants and ROS is essential for many physiological processes including spermatogenesis and sperm maturation [44, 45]. Studies have reported that GSH levels decline with aging and are accompanied by elevated oxidative stress in the heart, liver, kidneys, and brain [46, 47]. This is consistent with our results indicating that the GSH level decreased and ROS level increased in the testis of 16-month-old WT mice; however, these were observed when the *Gss*-deficiency mice were just 8 months old. *Gss* deficiency increased the age-related sensitivity of GSH and ROS balance. In 2-month-old S8/*Gss*^−/−^ mice, although *Gss* deficiency reduced GSH level, ROS was not elevated in the testes. This may be attributed to the self-regulation of the anti-oxidant system in the testis since we detected a compensatory increase in GPX4 level. With aging, the accumulation of ROS exceeds the range of testicular regulation, leading to severe testis injury in 8-month-old mice. The main phenotypes included disoriented testis structure, a large number of germ cells lost, and decreased sperm quality, which thus reduces fertility.

**Fig. 8:**
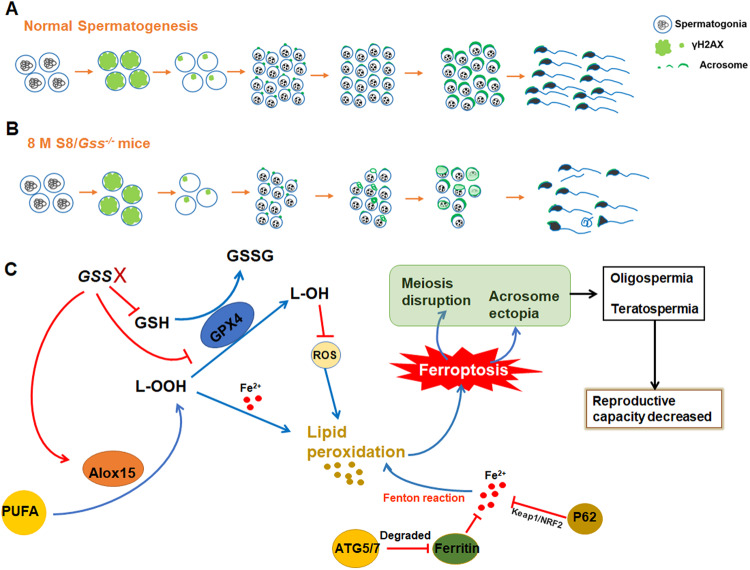
Schematic diagram of the *Gss* function during spermatogenesis in mice. **A** Illustration of spermatogenesis in normal mice. **B** Following *Gss* knockout in spermatogonia, spermatogenesis was disrupted in 8-month-old mice. **C** Molecular mechanism of testicular injury caused by *Gss* deficiency. The deficiency of *Gss* disrupts the balance of oxidation and anti-oxidant systems in the testis, which increases ROS level and lipid peroxidation, causing ferroptosis in the testis. Ferroptosis caused meiotic arrest and acrosomal dysplasia, eventually leading to oligospermia and teratospermia in mice, and significantly decreased fertility in male mice.

Our results suggested that postnatal deletion of *Gss* by Stra8-Cre has no effect on spermatogonia differentiation but plays an important role in meiosis and acrosome development during spermatogenesis. In normal mice, spermatocytes undergo two meiotic divisions into round spermatids, which morph into elongated sperms, accompanied by acrosome development (Fig. 8A). Following the deletion of *Gss* in germ cells for 8 months, meiosis could be initiated; however, the spermatocytes with punctate γH2AX were decreased (Fig. 8B). Consistently, the *Gss* was more abundantly expressed in such spermatocytes during spermatogenesis in WT mice, which also consistent with that GSH content is higher in pachytene spermatocytes [48]. This may due to that, the formation of programmed DNA double-strand breaks (DSBs) occurs at leptotene and zygotene during meiosis and is repaired by homologous recombination before late pachytene [30, 49, 50]. And sufficient GSH is essential to ensure the DSBs repaired and homologous recombination normally [51, 52]. Additionally, in 8-month-old S8/*Gss*^−/−^ mice, *Gss* deficiency caused the ectopic position of the acrosome of round spermatids and thus sperm malformation (Fig. 8B). A report has demonstrated, the decreased GSH induced by selenium deficiency diet causes spermatocyte and round spermatid reduction [53]. We concluded that in 8-month-old S8/*Gss*^−/−^ male mice, the disrupted meiosis and ectopic acrosome caused reduced sperm concentration and aberrant sperm morphology, significantly reducing fertility.

Next, the molecular mechanisms were explored. We found that *Gss* deficiency caused a reduction in GSH, inhibiting ROS removal in testis. It has been reported that ROS can act as signaling molecules to regulate antioxidant and pro-oxidant system, such as by activating or inhibiting NF-κB signal [54, 55]. Thereby, in the testis of S8/*Gss*^−/−^ mice, the accumulation of ROS may in turn affect regulatory proteins (for example, the NRF2 or NF-κB systems), resulting in decrease of anti-oxidant genes and increase of pro-oxidant genes. In the future, we will consider using RNA-sequencing and proteomics analysis between 8-month S8/Control and S8/*Gss*^−/−^ mice to explore the key factors and mechanisms that caused the imbalance of antioxidant and pro-oxidant systems in testes. As indicated in Fig. 8C, in the testes of 8-month-old S8/*Gss*^−/−^ mice, conditional knockout of *Gss* inhibiting GPX4 level and increasing ALOX15 expression. GPX4 used two molecules of GSH to reduce lipid hydroperoxide (L-OOH) to the corresponding hydroxy derivatives (L-OH), producing one molecule of GSSG [20]. This process was inhibited, causing an elevation in the ROS level and thus lipid peroxidation. To contrast, PUFAs produce peroxides in the presence of ALOX15 [38]. The increased ALOX15 expression promoted lipid peroxidation in the testes of 8-month-old S8/*Gss*^−/−^ mice. Additionally, it has been reported that autophagy-related proteins are also involved in ferroptosis. For instance, P62 reduces Fe^2+^ level and inhibits ferroptosis via the Kelch-like ECH-associated protein 1/nuclear factor erythroid 2-related factor 2 signaling pathway [56]; ATG5/7 increases Fe^2+^ level by degrading ferritin, thus, promoting ferroptosis [57]. Following *Gss* deficiency, the synthesis of GSH was reduced, oxidative and antioxidant was imbalanced, and autophagy was activated in the testis, all of which elevated testis ferroptosis.

We continued to explore a method to ameliorate the testis injury caused by *Gss* deficiency. Previous studies have reported that GSH improves sperm quality and testicular morphology in streptozotocin-induced diabetic mice [58]; Fer-1 reverses busulfan-induced testis ferroptosis by increasing GPX4 expressions [41]. In our study, both GSH and Fer-1 improved the fertility of older S8/*Gss*^−/−^ mice. This was indicated by a reduction in germ cell loss, mitigation of meiosis inhibition, reduction in malformed acrosome, and improved sperm quality. Despite the severe germ cell loss in 8-month-old S8/*Gss*^−/−^ mice, there was no reduction in the number of spermatogonia, which might be the main reason why the damage could be recovered. Additionally, GSH and Fer-1 increased GPX4 and inhibited the expression of ALOX5, thus, inhibiting lipid peroxidation and alleviating testis ferroptosis. These further suggested that supplementation of GSH or Fer-1 mitigated meiotic inhibition and acrosomal dysplasia owing to repress testis ferroptosis, further alleviating oligospermia and teratospermia in older mice.

This study provides new insights into ferroptosis. Ferroptosis is a time-dependent lesion triggered by the accumulation of ROS and Fe^2+^ over time in the state of injury. This is consistent with the previous studies reporting that Fe^2+^ plays an important role in various degenerative diseases [59]. Furthermore, ferroptosis is regulated by the combination of various signaling pathways and factors. As presented in the testes of 2-month-old S8/*Gss*^−/−^ mice, although the autophagy pathway, which can cause ferroptosis, was activated at this point, ferroptosis was not detected owing to the compensatory elevation of GPX4. This suggests a complementary relationship between the different pathways causing ferroptosis. Besides this, bilateral varicocele causes ferroptosis of human spermatozoa and affects semen quality in males with infertility [60]. In the future, whether fertility males accompanying testis or sperm ferroptosis will be worth exploring.

In this study, the relationship between ferroptosis and spermatogenesis was investigated. Conclusively, we observed that the deletion of *Gss* in germ cells for 8 months causes testis ferroptosis, which further leads to disrupted meiosis and disordered acrosome development, thus, reducing fertility in male mice. Furthermore, the administration of GSH and Fer-1 treatment to S8/*Gss*^−/−^ mice ameliorated testicular defects by inhibiting ferroptosis. The study indicated that supplementation with antioxidants or inhibition of ferroptosis may be a potential strategy to treat male infertility.

## Materials and methods

### Ethics statement

The experimental animals (CBA/J mice) were kept in a standard environment with a temperature of 22 ± 1 °C and a relative humidity of 50–60%. The treatment of all the animals was in strict accordance with the requirements of animal experiments at Shandong University (Jinan, Shandong, China), which were approved by the Ethics Committee of the School of Life Science, Shandong University. No blinding was done for animal studies.

### Generation of S8/*Gss*^−/−^ mice

*Gss*^flox/flox^ mice were generated using the clustered regularly interspaced short palindromic repeats (CRISPR)/Cas9 technology and targeted homologous recombination. Two guide RNAs (gRNAs) were designed for the left sides of the target exon 3 and two gRNAs for the right sides (gRNA1: 5′-TCTGCCAAGTTCCAGTTCGTTGG-3′, gRNA2: 5′-AAGGCGGGTTAGAT-CTAAGCGGG-3′, gRNA3: 5′-ATTTCCAACAACCAACGAACTGG-3′, gRNA4: 5′-GAAG-GCGGGTTAGATCTAAGCGG-3′). To generate the targeted conditional knockout offspring, the four gRNAs, Cas9 mRNA, the donor vector containing loxP sites, and the homology arm were co-injected into fertilized eggs. Polymerase chain reaction (PCR) was used to identify the F0 founder animals, and we further performed sequence analysis. The F0 founder mice were bred with wild-type (WT) mice for germline transmission. *Gss*^flox/flox^ or *Gss*^flox/-^ mice were crossed with Stra8-Cre mice to generate male mice with germ cell-specific knockout of the *Gss* gene. PCR analysis was used to identify the genotype of the mice with the primers listed as the following: the primers for flox insertion (F1: 5′-TTCTACCACCATAAACCCAACCTAC-3′; R1: 5′-GAGATATGCCTCCTGAGATTTCCA-3′); the primers for Cre (Cre F: 5′-TCGATGCAACGAGTGATGAG-3′; Cre R: 5′-TTCGGCTATACGTAACAGGG-3′).

### Fertility test

The reproductive ability of 2- and 8-month-old male mice was assessed by co-caging with adult WT females at the ratio of 1:2 for 3 months. Specifically, following treatment with drugs, the male mice were co-caged for 2 months to test their reproductive ability. Three mating cages were set up for each group and the number of pups was recorded.

### Histological analysis

The testes and epididymis were removed immediately from the mice and fixed in Animal Testicular Tissue Fixative (Servicebio, Wuhan, China) at room temperature overnight. The tissues were dehydrated with a series of ethanol concentrations, embedded in paraffin, and sliced. The thickness of the slice was controlled at 4 μm. Afterward, the samples were dried at 60 °C overnight. After being dewaxed and rehydrated, Hematoxylin and Eosin (H&E) were used to stain the slices following the standard procedures. Further, the slices were dehydrated in 90% and 100% gradient ethanol, and the slices were cleared with xylene and sealed with resin. The histological structure was observed under an optical microscope (Nikon YS100, Japan).

### Immunofluorescence staining

The testes of mice were fixed with 4% paraformaldehyde (PFA) overnight at 4 °C. Paraffin embedding and sectioning were then conducted using the usual procedures. The slices were dewaxed and rehydrated, immersed in 0.01% sodium citrate buffer (pH 6.0, Solarbio, Beijing, China), and boiled in water for 20 min. Next, the slices were permeated with 0.2% TrionX-100 at room temperature for 10 min. After being washed with phosphate-buffered saline (PBS), the samples were blocked with 5% goat serum at 37 °C for 30 min. Further, the sample was incubated with the primary antibodies at 4 °C overnight. The secondary antibodies conjugated to fluorescein isothiocyanate (FITC, 1:200; Invitrogen, USA) or tetramethylrhodamine isothiocyanate Fluor (TRITC, 1:200; Invitrogen, USA) were used to test the primary antibody. 4′,6-diamino-2-phenylindole (DAPI; Abcam, Cambridge, UK) was used to stain the nuclei. The samples were observed under the LSM900 confocal microscope system (Carl Zeiss AG, Jena, Germany), and the images were captured. The following antibodies were used: anti-γH2AX (1:200; Abcam, Cambridge, UK), anti-DEAD-box helicase 4 (DDX4) (1:200; Abcam, Cambridge, UK), anti-proliferating cell nuclear antigen (PCNA) (1:50; Santa, Texas, USA), anti-GSS (1:200; Affinity Biosciences, Jiangsu, China) antibodies.

### Analysis of sperm motility

The computer-assisted sperm analysis (CASA) system (Tsinghua Tongfang Co., Ltd., Beijing, China) was used to detect sperm concentration and motility. After being removed from mice, the caudal epididymis was cut in 500 µL M2 medium (Sigma-Aldrich, Missouri, USA) and placed at 37 °C under 5% CO_2_ for 30 min to allow the exit of sperms from the caudal epididymis. Ten microliters of sperm suspension were dropped on the cell counting plate and analyzed by the CASA system. Sperm concentration, forward motility, and no forward motility were recorded. More than 200 sperms per mouse were recorded, and three counting slides were measured and averaged.

### Sperm acrosome detection

The acrosomes were assessed by the FITC conjugated peanut agglutinin (PNA) (Sigama, Missouri, USA). After release from the caudal epididymis, the sperms were washed with PBS, dropped onto a glass slide, and dried. They were fixed with 4% PFA for 10 min and then stained with PNA (15 µg/ml in PBS) at 37 °C for 40 min. The paraffin sections of the testes were dewaxed and rehydrated, and PNA was further stained at 37 °C for 40 min. The sperm nuclei were stained by DAPI. Images were captured under the LSM900 confocal laser scanning microscope (Zeiss, Oberkochen, Germany). More than 200 sperms per mouse were counted; the number of normal and abnormal acrosomes was recorded independently by three researchers; the overall average number was calculated.

### Western blot

The total protein of testicular tissue was extracted by radioimmunoprecipitation assay (RIPA) lysis buffer (SparkJade, Shandong, China) with 1% phenylmethanesulfonyl fluoride (PMSF; Sigma-Aldrich, Shanghai, China). The protein of testis was separated using 10% or 12.5% sodium dodecyl sulfate-polyacrylamide gel electrophoresis (SDS-PAGE) and transferred to the polyvinylidene difluoride (PVDF) membranes (Millipore, Massachusetts, USA). The membranes were blocked with Protein Free Rapid Blocking Buffer (Shanghai Epizyme Biomedical Technology Co., Ltd, Shanghai, China) for 20 min and incubated with the primary antibodies at 4 °C overnight. After washing with TBST, the membranes were incubated with second antibodies at 37 °C for 1 h. The Spark ECL super kit (Sparkjade, Shandong, China) was covered on the membranes to present protein bands using Tanon 5200 Fully Automatic Chemiluminescence Image Analysis System. ImageJ software was used to quantify the bands on the membrane of Western blotting and normalized to β-actin. The following antibodies were used in this study: anti-β-actin (1:2000; Proteintech, Wuhan, China), anti-arachidonate 15-lipoxygenase (ALOX15) (1:1000; Affinity Biosciences, Jiangsu, China), anti-GPX4 (1:1000; Affinity Biosciences, Jiangsu, China), anti-cleaved-Caspase 3 (1:1000; Affinity Biosciences, Jiangsu, China), anti-Caspase 3 (1:1000; Affinity Biosciences, Jiangsu, China), anti-autophagy-related protein 5 (ATG5) (1:1000; Abcam, Cambridge, UK), anti-ATG7 (1:1000; Abcam, Cambridge, UK), anti-P62 (1:1000; Affinity Biosciences, Jiangsu, China), anti-microtubule-associated protein 1 A/1B light-chain 3 (LC3) (1:1000; Proteintech, Wuhan, China), anti-B-cell lymphoma (BCL)-2 associated X-protein (BAX) (1:1000; Affinity Biosciences, Jiangsu, China), anti-BCL-2 (1:1000; Affinity Biosciences, Jiangsu, China), anti-4-Hydroxynonenal (1:1000; Abcam, Cambridge, UK), and anti-3-Nitrotyrosine (1:1000; Abcam, Cambridge, UK) antibodies. The full length uncropped original western blots used in this manuscript are available in the “Original Western Blot Images” file.

### Scanning electron microscope

After washing with PBS, 10 µL of the sperm samples from the caudal epididymis of mice were dropped onto a glass slide and dried. The samples were then fixed with 2.5% glutaraldehyde. After being dehydrated in 25%, 50%, 75%, 90%, and 100% ethanol, the samples were subjected to critical point drying in an AUTOsamdri-815A (Tousimis, Maryland, USA). The samples were then sprayed with a layer of gold, and the sperm morphology was observed using the JEOL 7000 field emission gun scanning electron microscope (SEM) (QUANTA 250 FEG, FEI, USA).

### Immunohistochemistry (IHC)

For IHC, the testicular tissue was fixed with 4% PFA at 4 °C overnight. After being dehydrated in ethanol and embedded in paraffin, the testis was sliced, dewaxed, and rehydrated. The slices were immersed in 0.01% sodium citrate buffer (pH 6.0, Solarbio, Beijing, China) and boiled in water for 20 min. After the endogenous peroxidase was inhibited with H_2_O_2_ for 10 min at room temperature, the samples were incubated with 5% goat serum. Further, the primary antibodies were incubated at 4 °C overnight, and the secondary antibodies were incubated at 37 °C for 1 h. The SP-9000 SPlink Detection Kit (Biotin-Streptavidin HRP Detection Systems, ZSGB-BIO, Beijing, China) was used to detect antibody binding. The following antibodies were used: anti-SRY-box transcription factor 9 (1:200; ABclonal, Wuhan, China) and anti-c-KIT (1:200; R&D, Minnesota, USA) antibodies.

### Exogenous GSH and Fer-1 treatment

The 7-month-old S8/*Gss*^−/−^ mice were randomly divided into three groups. Two groups mice received an intraperitoneal injection of GSH or Fer-1 weekly for 12 weeks, and one group mice received with normal saline. Specifically, 0.1 mL of GSH (Sigma-Aldrich, Shanghai, China) was administered at a dose of 30 mg kg^-1^ body weight [58]. For exogenous Fer-1 (Shanghai Yuanye Bio-Technology Co., Ltd, Shanghai, China) treatment, mice were injected intraperitoneally with Fer-1 at a dose of 5 mg kg^-1^ body weight [61]. Bafilomycin A1 (Solarbio, Beijing, China) was administered every other day to mice by intraperitoneal injection at dose of 0.1 mg kg^-1^ body weight [62].

### Terminal deoxynucleotidyl transferase-mediated dUTP-biotin nick end labeling (TUNEL) assay

Two methods, TUNEL and 8-OHdG, were used to detect DNA damage. The TUNEL assay was used to examine DNA fragment breaks, and 8-OHdG was used to test DNA oxidative damage [40]. For the TUNEL assay, after being fixed with 4% PFA and embedded in paraffin, the testicular tissue was sliced. The slices were then cleared in xylene, and rehydrated. Further, the samples were incubated with 3% H_2_O_2_ in methanol solution for 10 min at room temperature and permeated with 0.1% TrionX-100 at 4 °C for 2 min. The apoptosis in the testis was detected by the TUNEL assay with the in situ Cell Death Detection Kit (Roche, Basel, Switzerland) according to the manufacturer’s instruction.

### 8-hydroxy-2′-deoxyguanosine (8-OHdG) concentration detection

After the testicular tissue was ground in normal saline on the ice, the samples were centrifuged at 13,000 rpm for 30 min. Then, 10 µL of the supernatant was dropped into the reaction pore of the Mouse 8-OHdG ELISA KIT (Y-S Bio-Technology, Shanghai, China) and mixed with 40 µL sample diluent. The sample was placed at 37 °C for 1 h. According to the manufacturer’s protocols of the 8-OHdG ELISA KIT, the absorbance of the sample at 450 nm was measured on Microplate Reader (VICTOR3, PerkinElmer, Finland), and the concentration was calculated.

### Tissue Fe^2+^ content detection

The collected testicular tissues were ground in normal saline and centrifuged at 13,000 rpm for 30 min. Afterward, the supernatant was collected. The Fe^2+^ content in the supernatant was then detected using the Tissue Iron Assay Kit (Nanjing Jiancheng Bioengineering Institute, Nanjing, China) according to the manufacturer’s instruction. The content was calculated by measuring the absorbance of the sample on Microplate Reader (VICTOR3, PerkinElmer, Finland).

### Detection of GSH concentration

After being ground in normal saline on the ice, and then the mixture was centrifuged at 13,000 rpm for 30 min to collect the supernatant. The total GSH content was calculated following a series of experiments according to the GSH Assay Kit (Beyotime, Shanghai, China).

### ROS detection

The mouse sperm was collected in the same way for each group of mice, and 2′,7′-dichlorodihydrofluorescein diacetate (DCFH-DA) from Reactive Oxygen Species (ROS) Assay Kit (Beyotime, Shanghai, China) was added to the samples. After incubating at 37 °C for 20 min, the samples were centrifuged for 5 min at 2600 rpm; further, the supernatant was discarded and the pellet was resuspended with PBS. The testicular tissue was cut in saline, and the cells were separated. Next, DCFH-DA was added to the samples incubating at 37 °C for 20 min. The cells were separated again and resuspended with PBS. Microplate Reader (VICTOR3, PerkinElmer, Finland) was used to detect the fluorescence signal at an excitation wavelength of 488 nm.

### Quantitative reverse-transcription PCR (qRT-PCR)

For qRT-PCR, the total RNA of testis was extracted by TRIzol reagent (Invitrogen, Carlsbad, USA). The reverse transcriptase (Toyobo, Osaka, Japan) was used to reverse-transcribe the RNA sample. The Bio-Rad Sequence Detection System (Bio-Rad Laboratories Inc., California, USA) was used to detect the expression levels of Gpx4, *glutathione-disulfide reductase* (*Gsr*), glutaredoxin (*Glrx)*, *NADPH quinone oxidoreductase* (*Nqo1*), *Alox15*, and *lactoperoxidase* (*Lpo*) mRNA using qRT-PCR. The primers were as follows: primers for *Gpx4* (forward: 5′-GCAACCAGTTTGGGAGGCAGGAG-3′; reverse: 5′-CCTCCATGGGACCATAGCGCTTC-3′), primers for *Gsr* (forward: 5′-TATGTGAGCCGCCTGAACA-3′; reverse: 5′-GTGGCAATCAGGATGTGTGG-3′), primers for *Glrx* (forward: 5′-GCTCAGGAGTTTGTGAACTGC-3′; reverse: 5′-AGAAGACCTTGTTTGAAAGGCA-3′), primers for *Nqo1* (forward: 5′- ACTTCAACCCCATCATTTCCAG-3′; reverse: 5′-TATCACCAGGTCTGCAGCTT-3′), primers for *Alox15* (forward: 5′-GACTTGGCTGAGCGAGGACT-3′; reverse: 5′-CTTGACACCAGCTCTGCA-3′), primers for *Lpo* (forward: 5′-CTGGACCAGAAGAGATCCATG-3′; reverse: 5′-TCACCAGGTGGGAACATGATGG-3′), and primers for *Actb* (forward: 5′-CGGTTCAGGTACTCAGTCAGTCATCC-3′; reverse: 5′-GGTGGGGTCATGTGTGTGG-3′). The relative mRNA level was calculated by the 2^−ΔΔCt^ method and normalized to *Actb*.

### Statistical analysis

Experimental sample size was estimated based on our past experience performing similar studies evaluating rescue efficiency in mice. There was no data exclusion. All experimental data were represented as mean ± SD, and the student’s t-test was used to compare the data, with *P* < *0.05* considered statistically significant. These graphs were created by GraphPad Prism software. More than three replicates were conducted to ensure reproducibility. The sample sizes and the defined replicates are displayed in the figure legends.

### Reporting summary

Further information on research design is available in the Nature Research Reporting Summary linked to this article.

### Supplementary information

Supplementary Figures and Legends

Reporting summary

Original Data File

## Data Availability

The datasets generated during and analysed during the current study are available from the corresponding author on reasonable request. Supplementary information is available at *Cell Death & Disease*’s website.
