# Supplementary material for: Gss deficiency causes age-related fertility impairment via ROS-triggered ferroptosis in the testes of mice
Source: Cell Death Dis. 2023 Dec 19;14(12):845. doi: 10.1038/s41419-023-06359-x (PMC10730895; doi:10.1038/s41419-023-06359-x)
Supplement: Supplementary file 1 — Supplementary Figures and Legends [file 41419_2023_6359_MOESM1_ESM.pdf]

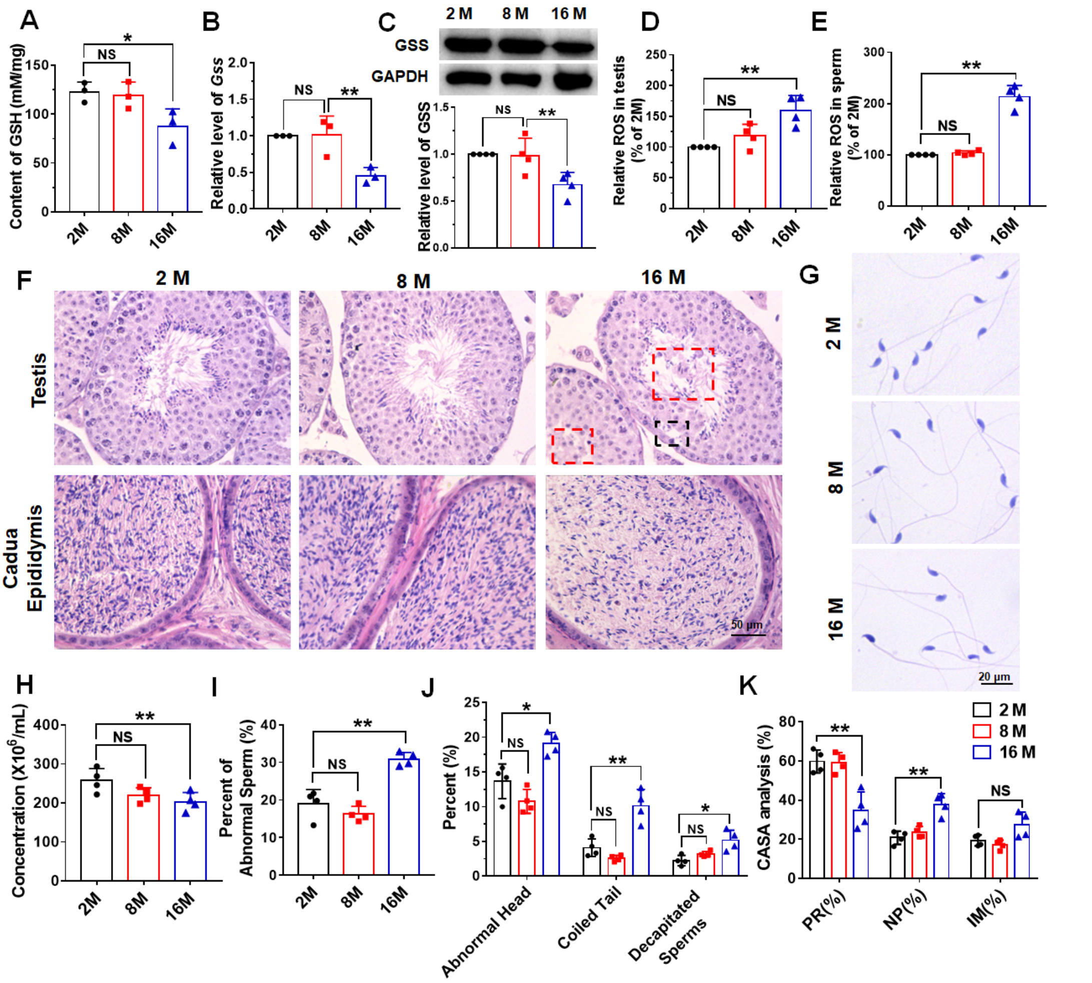

Figure S1. ROS level was increased and sperm quality was decreased in aged mice

A Levels of GSH in the testes of WT mice at 2, 8, and 16 months. $*P < 0.05$; NS, non-significant; n = 3 (biologically independent animals). **B, C** The mRNA and protein levels of *Gss* in the testes of WT mice at different ages. $**P < 0.01$; NS, non-significant; n = 3 or 4 (biologically independent animals). **D, E** The relative levels of ROS in the testes or sperms from 2-, 8-, and 16-month-old WT mice. $**P < 0.01$; NS, non-significant; n = 4 (biologically independent animals). **F** The H&E staining results of testis and caudal epididymis of mice at different ages. Scar bar = 50 μm. **G** H&E staining of sperms from caudal epididymis of 2-, 8-, and 16-month-old WT mice. Scar bar = 20 μm. **H** Sperm concentrations under the same treatment condition of WT mice. **I, J** Statistical results of sperm malformation. **K** The CASA analysis to detect sperm motility in WT mice at different ages. **H-K** $*\ P < 0.05$, $**\ P < 0.01$; NS means non-significant; n = 4 (biologically independent animals).

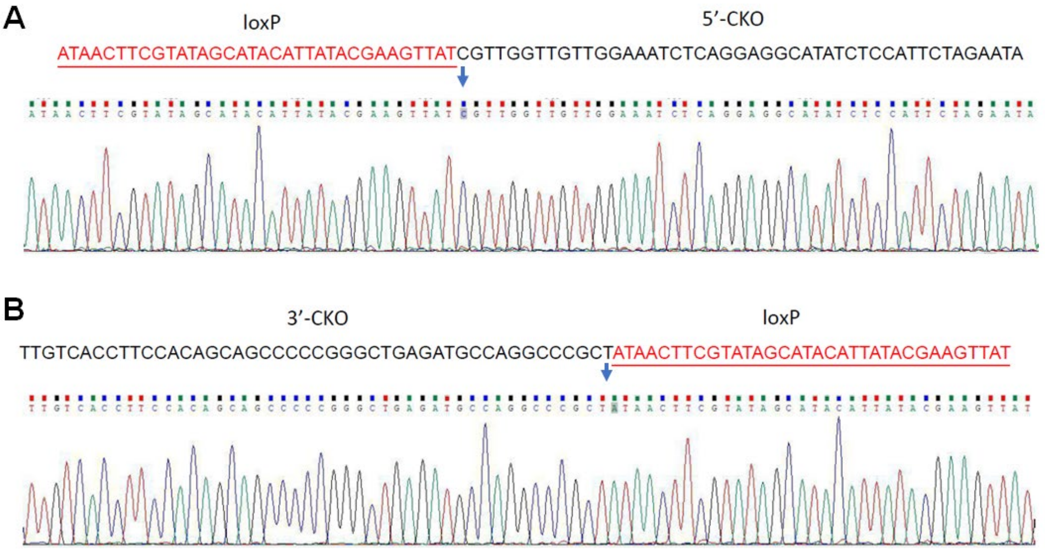

Figure S2 Sequencing results of homozygous mice

The sequencing results of *Gss* $^{Flox/Flox}$ mice by primers F1, R1 **A** and F2, R2 **B**.

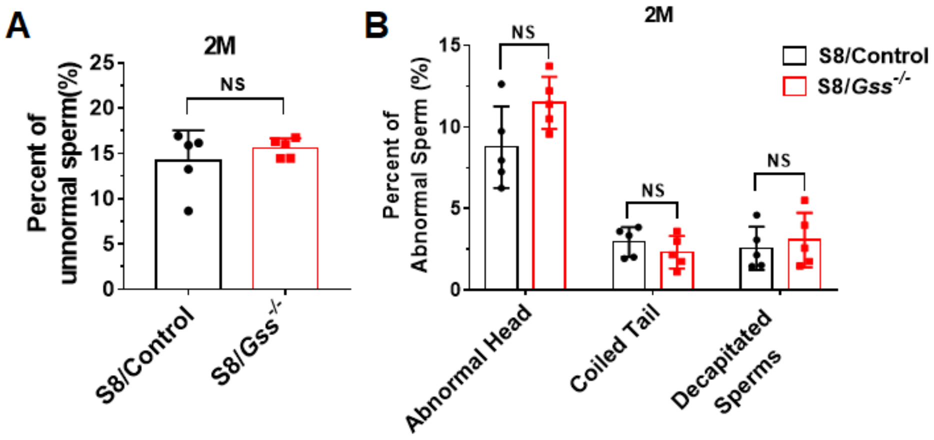

Figure S3 Statistical results of abnormal sperms in 2-month-old mice

A The percentage of sperms with abnormal morphology, and **B** the proportion of various types of abnormalities in 2-month-old mice. NS indicates non-significant, n = 5 (biologically independent animals).

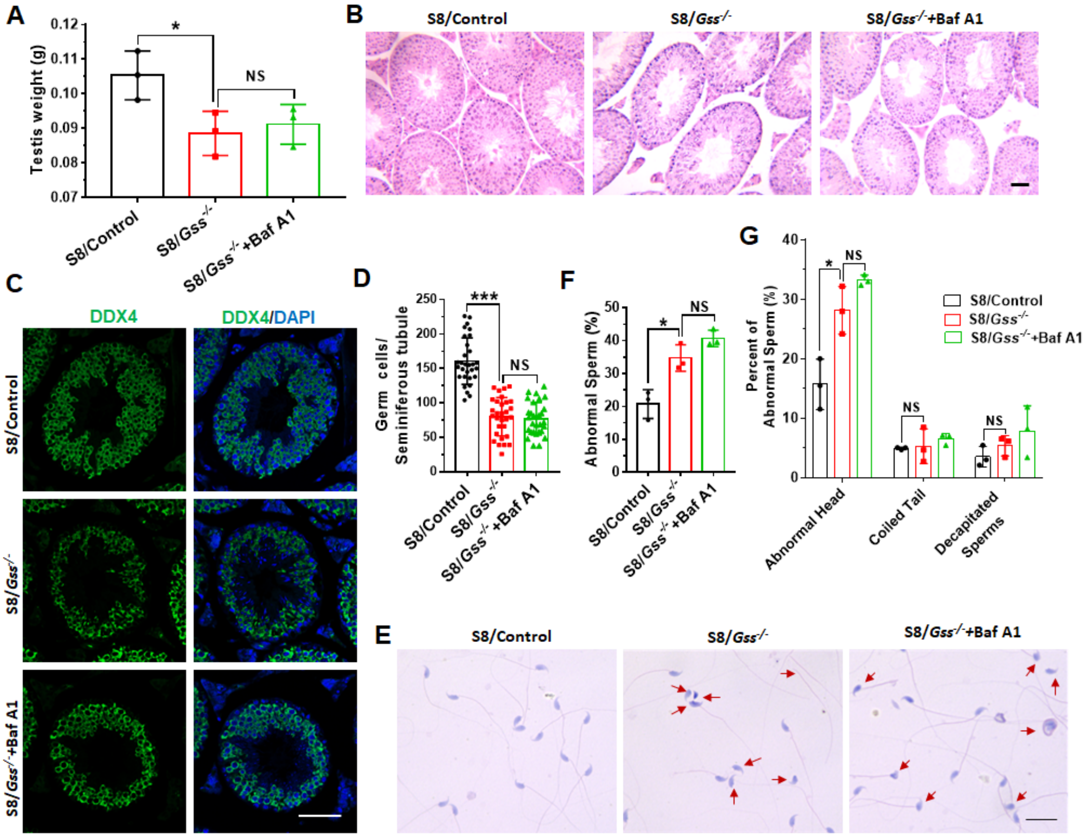

Figure S4 The effects of Bafilomycin A1 in testis and sperms of 8-month-old S8/*Gss*$^{-/-}$ mice

A The weight of testis from S8/Control, S8/*Gss*$^{-/-}$ mice, S8/*Gss*$^{-/-}$ + Bafilomycin A1 (Baf A1) mice. **P < 0.05*; NS, non-significant; n = 3 (biologically independent animals). **B** H&E staining showed the morphology of testis after treatment by Baf A1. Scar bar = 50 μm. **C** The immunofluorescence of DDX4 to mark the germ cells in testis. Scar bar = 50 μm. **D** Statistical results of the number of germ cells in single seminiferous tubule. ****P < 0.001*; NS, non-significant; n = 3 (biologically independent animals). **E** The H&E staining of sperms from cauda epididymidis from mice. Scar bar = 20 μm. The red arrow points to the malformed sperm. **F, G** Percentage of total abnormal sperms and percentage of abnormal sperms in different types. **P < 0.05*; NS, non-significant; n = 3 (biologically independent animals).
