# Supplementary figures and images for: Gss deficiency causes age-related fertility impairment via ROS-triggered ferroptosis in the testes of mice
Source: Cell Death Dis. 2023 Dec 19;14(12):845. doi: 10.1038/s41419-023-06359-x (PMC10730895; doi:10.1038/s41419-023-06359-x)

**Original Western blot images**


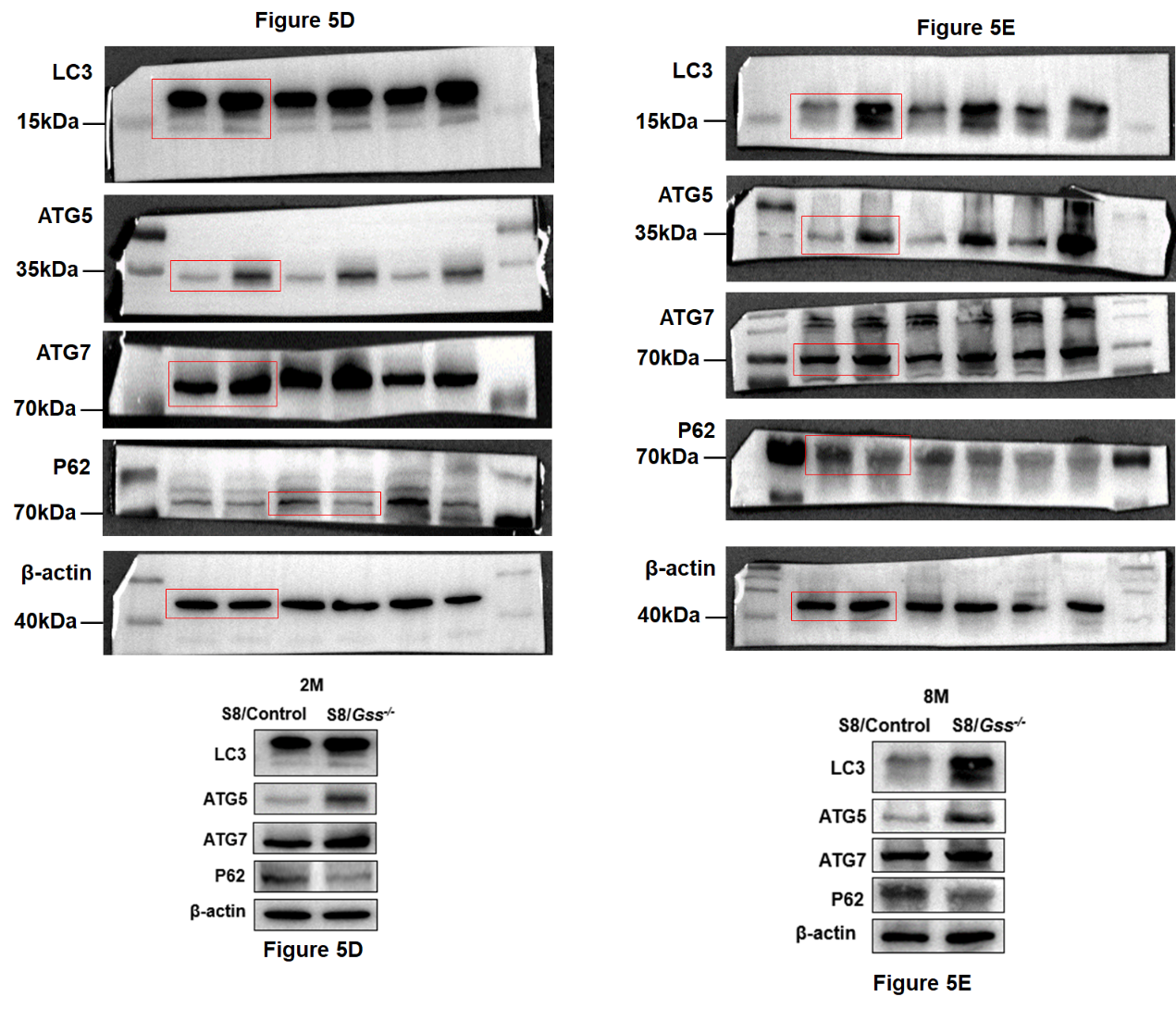

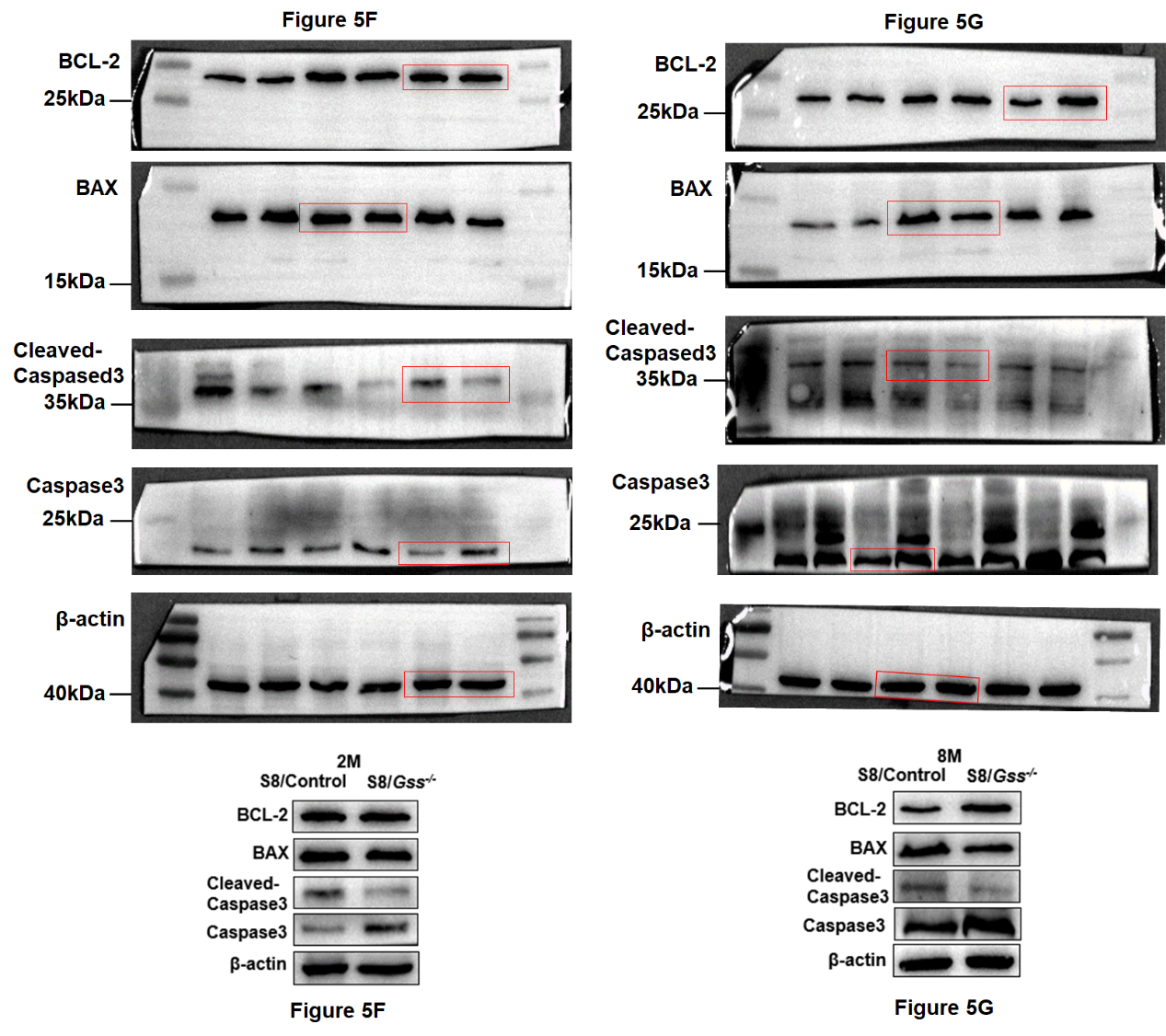

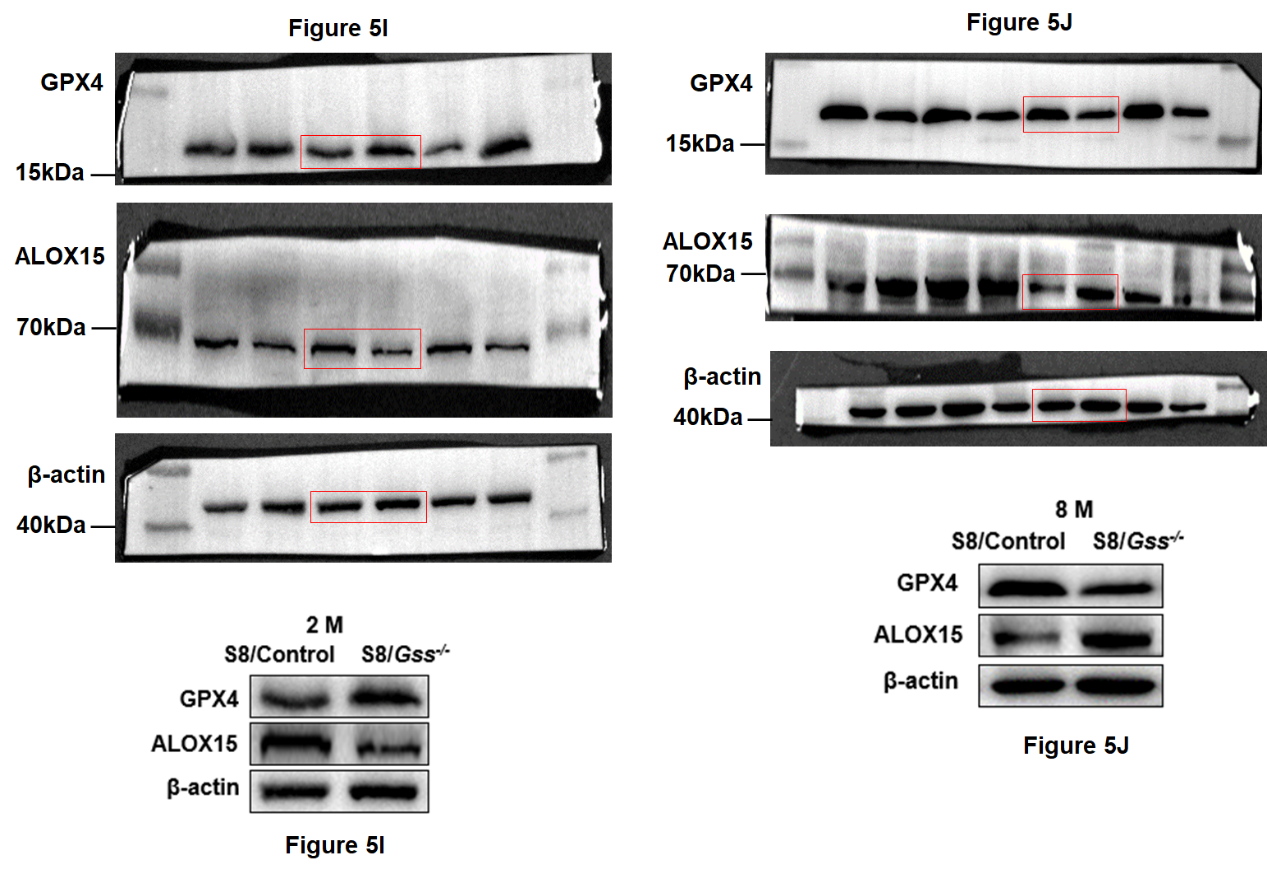

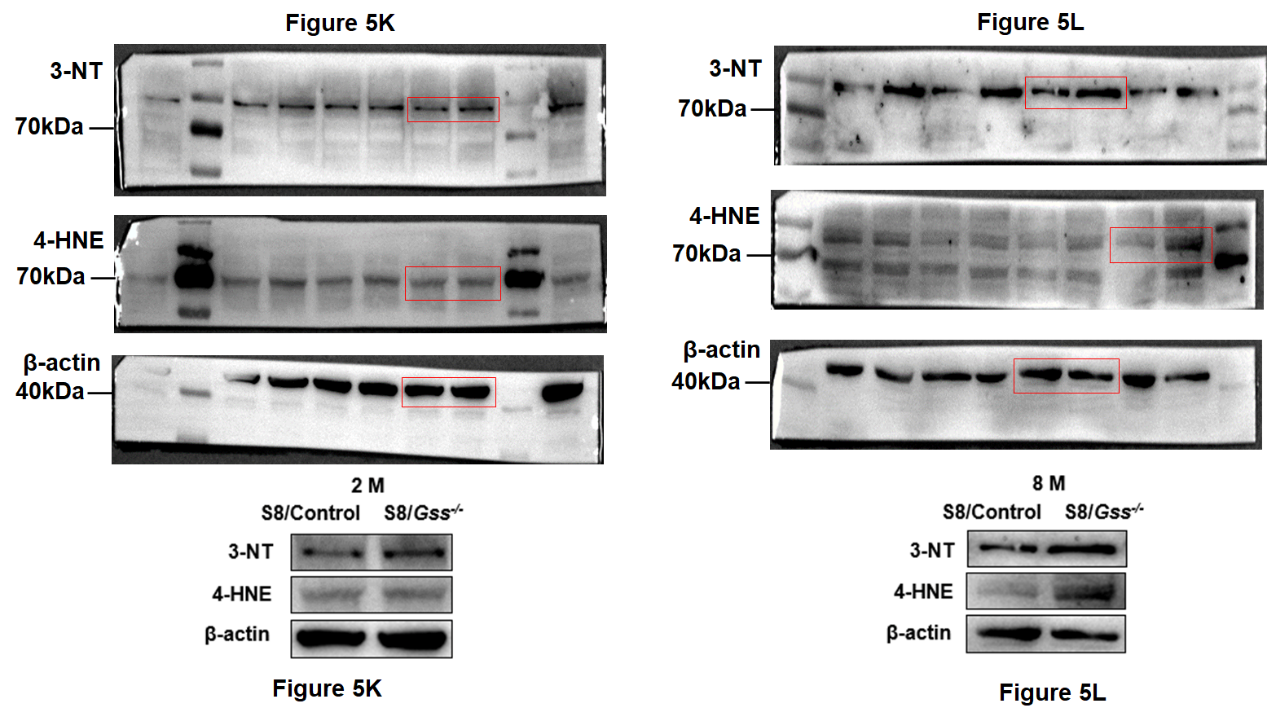

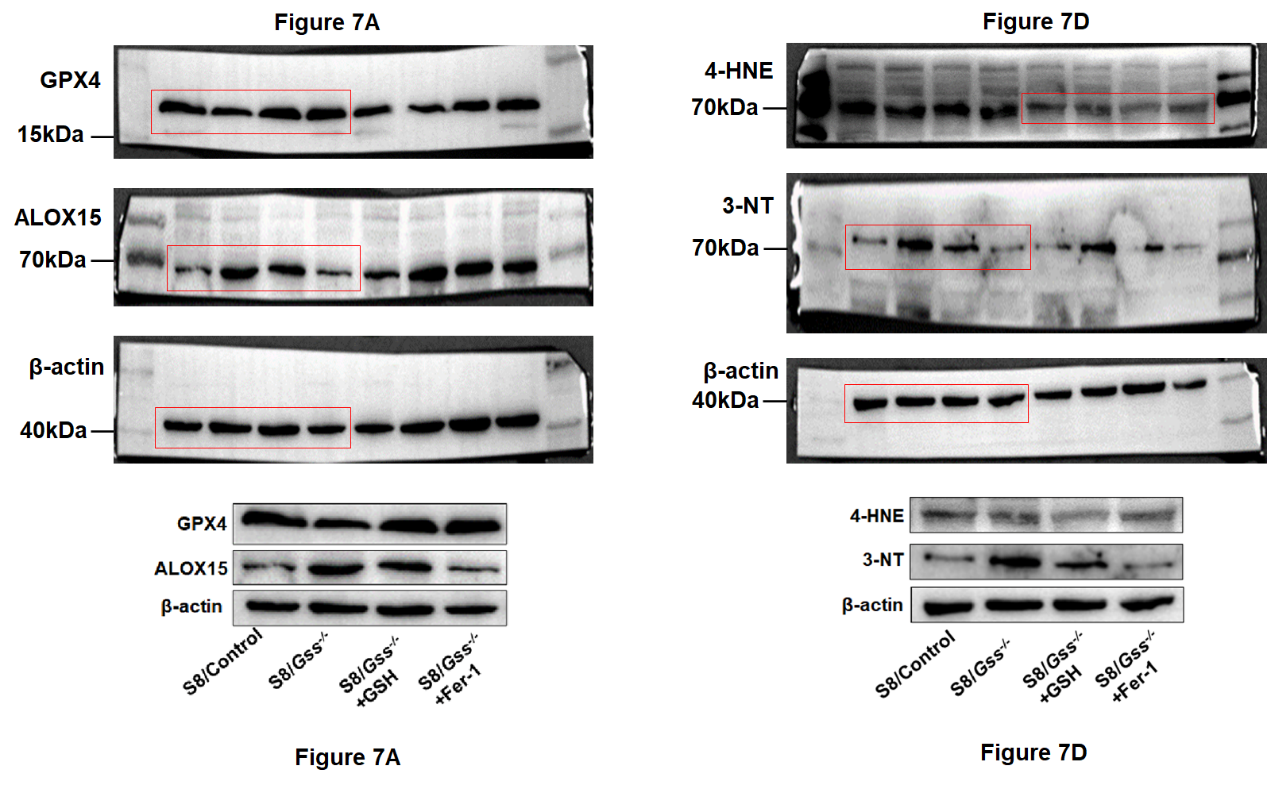


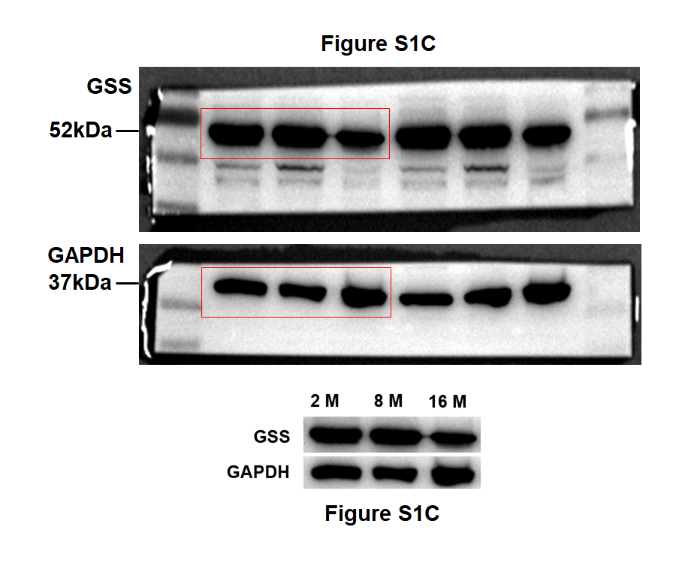

Supplement: Supplementary file 3 — Original Data File [file 41419_2023_6359_MOESM3_ESM.docx]
